# DcR3 combined with hematological traits serves as a valuable biomarker for the diagnosis of cancer metastasis

**DOI:** 10.18632/oncotarget.22544

**Published:** 2017-11-20

**Authors:** Junxin Li, Ni Xie, Jianhui Yuan, Lvyan Liu, Qiming Zhou, Xiaohu Ren, Qian Chen, Guizhong Zhang, Qingguo Ruan, Youhai H. Chen, Xiaochun Wan

**Affiliations:** ^1^ Shenzhen Laboratory of Fully Human Antibody Engineering, Institute of Biomedicine and Biotechnology, Shenzhen Institutes of Advanced Technology, Chinese Academy of Sciences, University City of Shenzhen, Xili Nanshan, Shenzhen, 518055, P.R. China; ^2^ University of Chinese Academy of Sciences, Beijing, 100049, P.R. China; ^3^ Institute of Translation Medicine, Shenzhen Second People's Hospital, Shenzhen, 518035, P.R. China; ^4^ Institute of Toxicology, Shenzhen Center for Disease Control and Prevention, Shenzhen, 518055, P.R. China; ^5^ Department of Oncology, Nanshan Hospital of Shenzhen, Shenzhen, 518055, P.R. China; ^6^ Department of Pathology and Laboratory Medicine, University of Pennsylvania School of Medicine, Philadelphia, PA, 19104, USA

**Keywords:** DcR3, PDW, HGB, HCT, metastasis

## Abstract

Decoy receptor 3 (DcR3) is abnormally up-regulated in many cancer cells. It may help cancer cells to escape from immune surveillance and establish metastatic lesions. However, whether DcR3 can be used as a biomarker for the diagnosis of cancer metastasis is unclear. In this study, sera from healthy controls and patients with different cancers were collected, and tested for their DcR3 levels by ELISA. Significantly elevated DcR3 levels were observed in the sera of patients with gastric cancer (2.04 ± 1.01, *P* = 0.0061), lymphoma (1.62 ± 0.75, *P* = 0.041), and breast cancer (1.53 ± 0.51, *P* = 0.023). DcR3 was found to be a suitable biomarker for identifying gastric cancer patients. Importantly, DcR3 was positively associated with platelet distribution width (PDW) (*P* = 2.45 × 10^−6^, *R* = 0.63) in metastatic cancers but negatively associated with hemoglobin (HGB) (*P* = 0.002, *R* = −0.59) and hematocrit (HCT) (*P* = 0.001, *R* = −0.62) in non-metastatic cancers. Combined with PDW, HGB and HCT, serum DcR3 could be used to predict the occurrence of cancer metastasis. These findings indicate that DcR3 could be used as a biomarker for the diagnosis of gastric cancer, and for cancer metastasis in combination with hematological traits.

## INTRODUCTION

Decoy receptor 3 (DcR3), also known as tumor necrosis factor receptor superfamily member 6B (TNFRSF6B), is a soluble protein that competitively binds Fas ligand [1]. DcR3 has an anti-apoptotic role and is highly expressed in malignant tumors, such as gastric carcinomas [2], liver cancer [3], and breast infiltrating ductal carcinoma (IDC) [4]. DcR3 has been found to be a pleiotropic immune-modulator, and was proposed to serve as a biomarker for inflammatory diseases and cancer [5, 6]. It was also considered to be a novel prognostic biomarker for small intestinal neuroendocrine tumors [7]. In addition, a considerable amount of evidence suggests that serum DcR3 may have a predictive value for stage pN2 and the prognosis (TNM classification) of both pancreatic carcinoma and gastric cancer [8, 9]. Recently, we found that DcR3 promoted hepatoma cell migration by downregulating E-cadherin expression [10]. Therefore, DcR3 expression may also be closely related to cancer metastasis.

Hematological traits are important clinical parameters and closely related to malignancies such as colorectal cancer [11]. Platelet distribution width (PDW) is an index describing variations in platelet size and is used in differential diagnoses of thrombocytopenia. In recent years, increasing studies have shown that PDW is a diagnostic or prognostic indicator for various cancers, such as colorectal cancer [12], gastric cancer [13], and thyroid cancer [14]. It was reported that the combined detection of PDW and CEA is valuable in differentiating gastric cancer from gastric ulcer and controls [15]. Hematocrit (HCT) is the percentage of red blood cells in blood by volume. Abnormally reduced HCT and hemoglobin (HGB) levels can indicate anemia in patients [16]. Since anemia is closely related to a worse prognosis in cancer patients [17], HCT and HGB could be used as prognostic parameters [16, 18]. Nevertheless, the association between hematological traits and cancer metastasis has not been studied.

Metastasis is a major cause of mortality in cancer patients. Tumor cells must escape from immune surveillance in cancer metastasis [19, 20]. DcR3 is a well-known immune suppressor [21, 22], and abnormally elevated DcR3 may help cancer cells metastasize by suppressing immune responses. However, whether DcR3 is valuable in differentiating cancer metastasis from non-metastasis remains largely unclear. In this study, we measured serum levels of DcR3 in several types of cancer and analyzed the correlation between DcR3 and hematological traits. Based on these observations, we further evaluated the value of DcR3 in detecting cancer metastasis in combination with hematological traits.

## RESULTS

### Serum level of DcR3 was elevated in three types of human cancers

**Figure 1:**
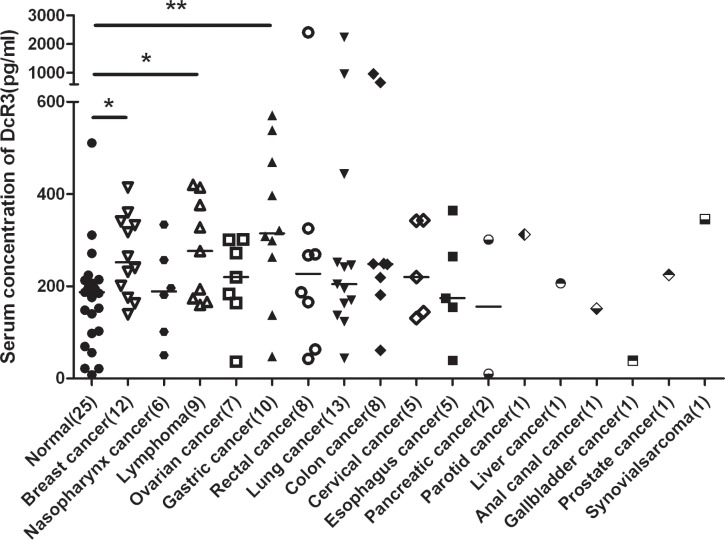
Serum DcR3 levels in cancer patients DcR3 was significantly elevated in gastric cancer, lymphoma and breast cancer. Median DcR3 levels are indicated by short bars. The number of patients tested (*n*) is shown. ^*^compared with healthy controls, *P* < 0.05. ^**^compared with healthy controls, *P* < 0.01.

To explore diagnostic application based on DcR3 expression, we established a sensitive ELISA to measure DcR3 serum levels (11–12000 pg/ml, *R*^2^ = 0.9941). The results showed significant elevations of DcR3 in gastric cancer (2.04 ± 1.01, *P* = 0.0061), lymphoma (1.62 ± 0.75, *P* = 0.041), and breast cancer (1.53 ± 0.51, *P* = 0.023), but not in other cancers tested (Figure 1).

### ROC analysis suggested DcR3 was a valuable biomarker for identifying gastric cancer

**Figure 2:**
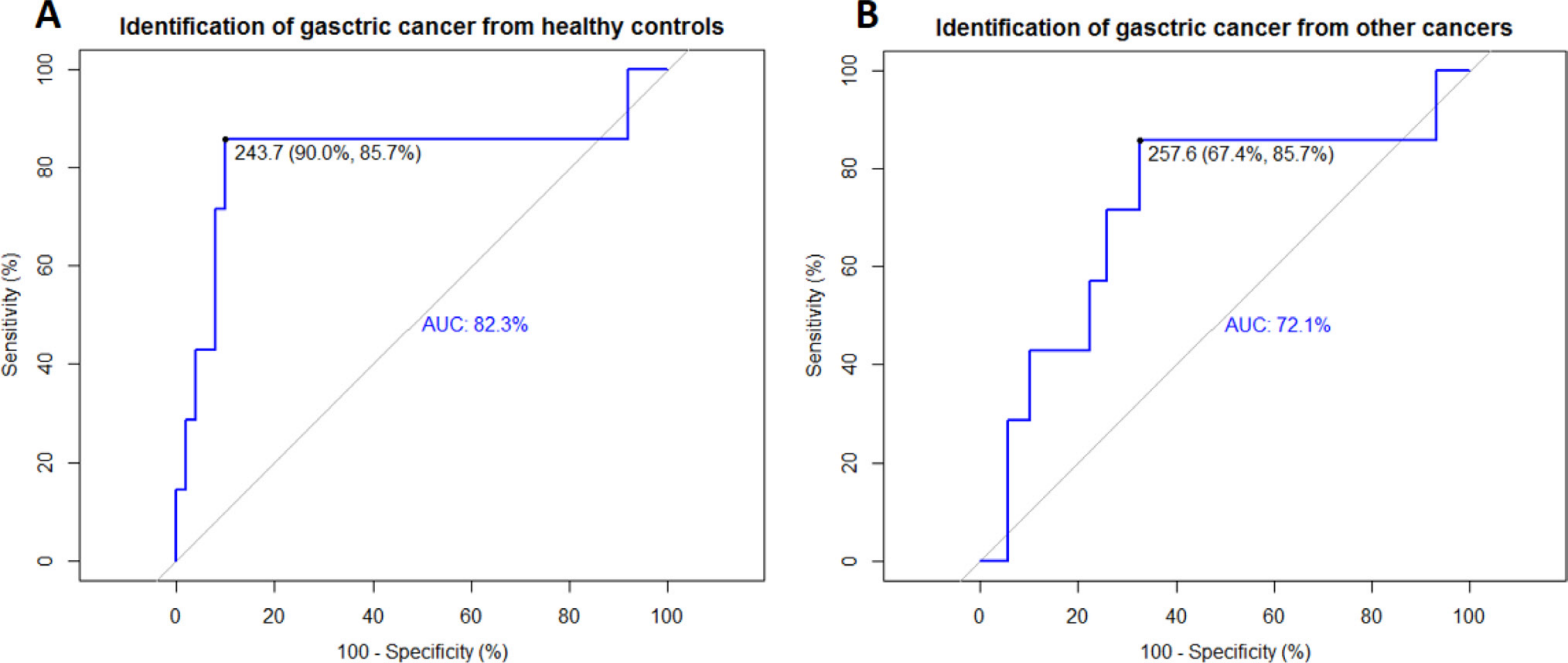
ROC curves revealed the diagnostic ability of DcR3 for gastric cancer Identifications of gastric cancer from healthy controls and other cancers by DcR3 were evaluated using ROC curves. (**A**) Identification of gastric cancer from healthy controls using the ROC curve; sensitivity = 85.7%, specificity = 90.0%, AUC = 82.3%, threshold = 243.7 pg/mL; (**B**) Identification of gastric cancer from other cancers using the ROC curve; sensitivity = 85.7%, specificity = 67.4%, AUC = 72.1%, threshold = 257.6 pg/mL.

The data of serum concentrations of DcR3 were analyzed using the R package “*pROC*.” The results suggested high specificity, sensitivity, and accuracy for distinguishing gastric cancer patients from healthy controls (Figure 2A). Further analysis revealed that DcR3 was robust enough even to distinguish gastric cancer from other cancers (Figure 2B).

### DcR3 is associated with hematological traits

**Figure 3:**
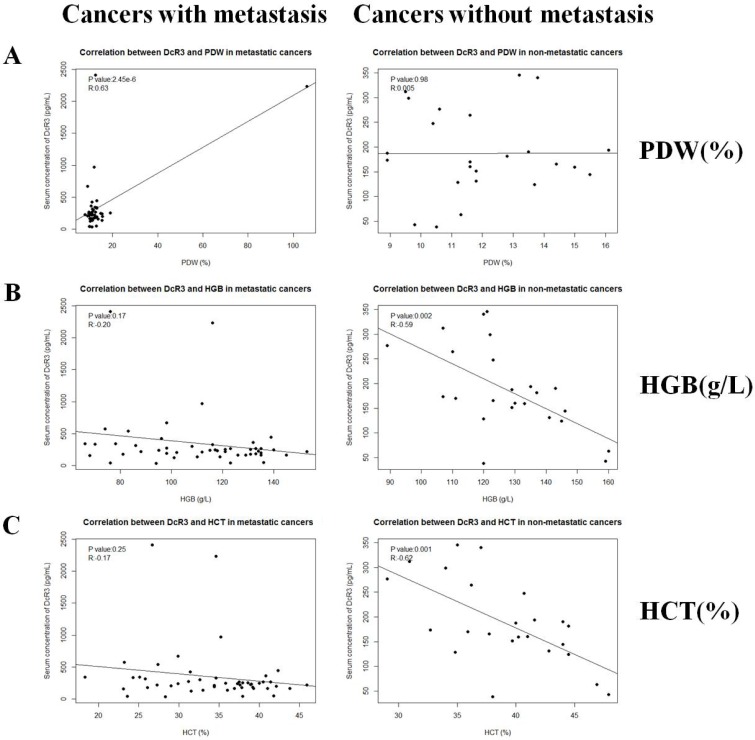
Correlations between serum DcR3 and hematological traits Serum DcR3 level was associated with PDW, HGB and HCT. (**A**) Serum DcR3 level was positively correlated with PDW (*R* = 0.627, *P* = 2.45 × 10^−6^) in metastatic cancers while not correlated with PDW (*R* = 0.0049, *P* = 0.98) in non-metastatic cancers; (**B**) Serum DcR3 level was not correlated with HGB (*R* = −0.20, *P* = 0.17) in metastatic cancers while negatively correlated with HGB (*R* = −0.59, *P* = 0.002) in non-metastatic cancers; (**C**) Serum DcR3 level was not correlated with HCT (*R* = −0.17, *P* = 0.25) in metastatic cancers while negatively correlated with HCT (*R* = −0.62, *P* = 0.001) in non-metastatic cancers.

ELISA and routine blood test data were organized and then analyzed using the Spearman correlation test. As analyzed in Figure 1, compared with healthy controls, many types of cancer had no significant differences in the serum DcR3 levels, of which metastatic cancers did. Because of the limited sample size for each individual type of cancer, we divided all of the cancer cases into metastatic and non-metastatic groups. Fifty-eight metastatic and Thirty-two non-metastatic cases were included. Clinical data of cancer patients are shown in Table 1. The results indicated that DcR3 was positively associated with PDW (*P* = 2.45 × 10^−6^, *R* = 0.63) in subjects with metastatic cancers (Figure 3A). Additionally, DcR3 was found to be negatively associated with HGB (*P* = 0.002, *R* = −0.59) and HCT (*P* = 0.001, *R* = −0.62) in subjects with non-metastatic cancers (Figure 3B, 3C). The correlations among PDW, HCT and HGB are shown in Supplementary Figure 2.

**Table 1:** Clinical and laboratory characteristics of the participants

Variables	Metastasis (*n* = 58)	Non-metastasis (*n* = 32)	*P*-value
Breast cancer (*n*)	9	3	NA
Lung cancer (*n*)	9	4	NA
Colon cancer (*n*)	6	2	NA
Gastric cancer (*n*)	7	3	NA
Rectal cancer (*n*)	5	3	NA
Lymphoma (*n*)	3	6	NA
Ovarian cancer (*n*)	5	2	NA
Cervical cancer (*n*)	3	2	NA
Esophagus cancer (*n*)	3	2	NA
Nasopharynx cancer (*n*)	3	2	NA
Other cancers (*n*)	5	3	NA
Ages (years)	51.5 (12.2)	47.4 (15.5)	0.102
Gender (males,%)	25 (43.9)	12 (44.4)	0.056
DcR3 (pg/ml)^*^	335.6 (413.6)	228.5 (173.7)	<0.05
PDW (%)	14.1 (13.9)	11.8 (1.9)	0.058
Haemoglobin (g/dl)^*^	112.2 (24.1)	124.5 (16.7)	<0.05
Hematocrit (%)^*^	34.6 (6.8)	38.1 (5.1)	<0.05
DcR3 × PDW ÷ (HGB × HCT)^***^	1.4 (1.9)	0.4 (0.2)	<0.001

Data are presented as means(SD). *P*-Value was calculated by chi-square test (Age) or Mann-Whitney *U* test. Metastasis includes lymph node metastasis and distant metastasis. The number of patients tested (*n*) is shown.

^*^compared with non-metastatic cancers, *P* < 0.05.

^***^compared with non-metastatic cancers, *P* < 0.001.

### The combination of PDW, HGB, and HCT improves the detective ability of DcR3 for tumor metastasis

**Figure 4:**
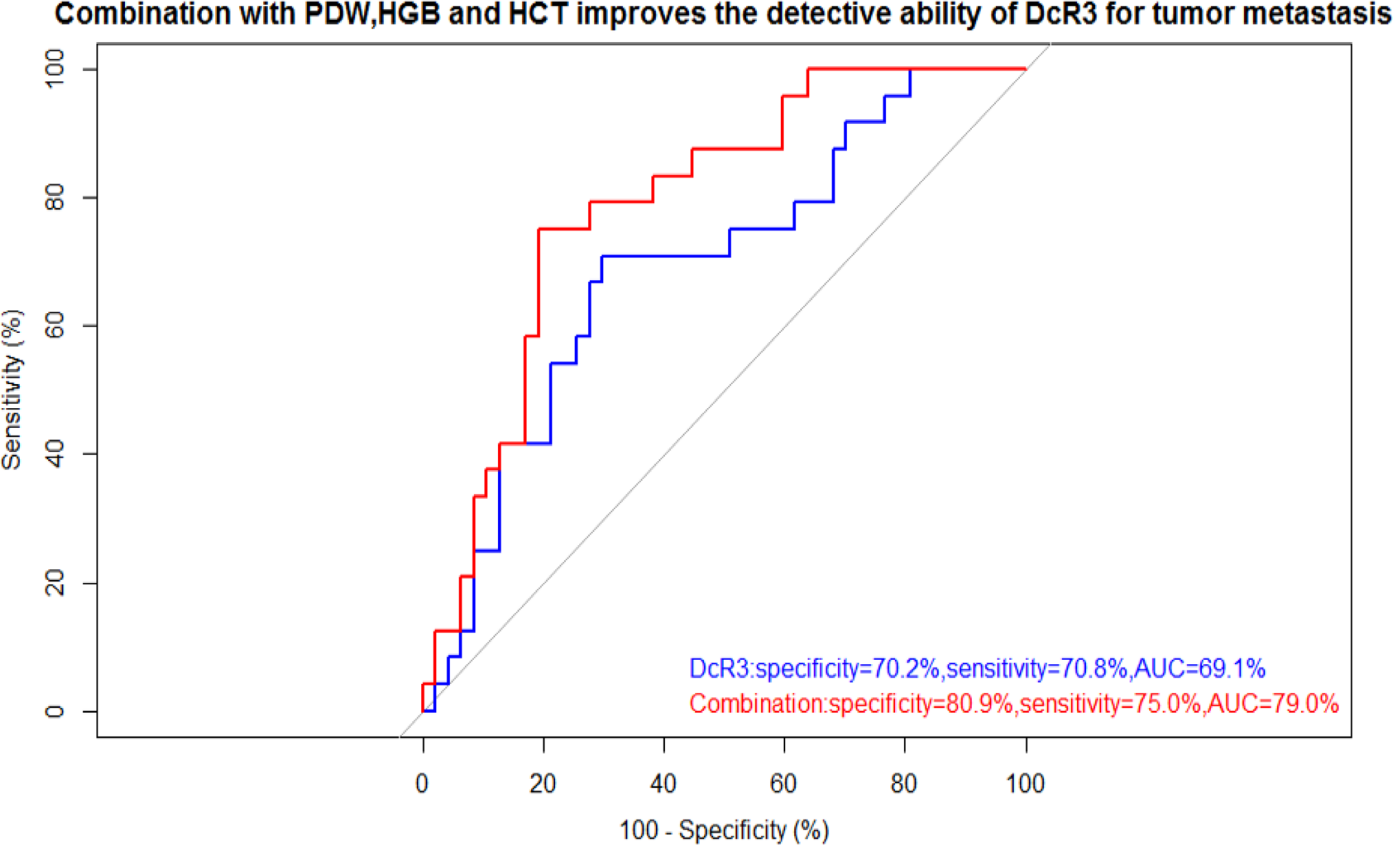
ROC curve showed the utility of alone or combination for the diagnosis of tumor metastasis Combined use of PDW, HGB, HCT and DcR3 improves both specificity and sensitivity for the diagnosis of tumor metastasis. The thresholds of DcR3 alone and combination were 194.30 pg/ml and 0.54, respectively.

The correlation analysis indicated that DcR3 was positively associated with PDW and negatively associated with HGB and HCT. Thus, to improve the diagnostic power of DcR3, we tried different mathematical combinations of DcR3, PDW, HGB and HCT, including Equation 1, Equation 2, Equation 3 and Equation 4. The results suggested the combination with best performance is the one shown in Equation 1. The novel indicator (specificity: 80.9%, sensitivity: 75.0%, AUC: 79.0%) showed with better specificity, higher sensitivity, and greater accuracy than DcR3 alone (specificity: 70.2%, sensitivity: 70.8%, AUC: 69.1%) (Figure 4). As shown in Table 2, the novel indicator was more strongly associated with metastatic risk (OR: 10.39, 95% CI: 3.27–22.10). The results of ROCs and ORs of other Equations are shown in Supplementary Figure 1 and Supplementary Table 1.

**Table 2:** Metastasis risk according to DcR3 and the novel indicator

Parameter	DcR3 (pg/ml)				DcR3 × PDW ÷ (HGB × HCT)			
	>194.30	<194.30	OR (95% CI)	*P*-value	>0.54	<0.54	OR (95% CI)	*P*-value
Metastasis (*n* = 58)	*n* = 44	*n* = 14	5.23 (1.94–11.85)	0.001	*n* = 49	*n* = 9	10.39 (3.27–22.10)	2.17 × 10^−5^
Non-Metastasis (*n* = 32)	*n* = 12	*n* = 20			*n* = 11	*n* = 21		

The number of patients tested (*n*) is shown.

OR: Odds Ratio.

CI: Confidence Interval.

The novel indicator = DcR3 × PDW ÷ (HGB × HCT) Equation 1

## DISCUSSION

Even in the face of the best currently available medical and surgical treatments, the overall prognosis of patients with metastatic cancers remains poor. In this study, we collected serum samples from patients with different caners and analyzed the DcR3 level. Results indicated an abnormal elevation of DcR3 in gastric cancer, lymphoma, and breast cancer. Results also suggested that DcR3 was a candidate biomarker for the highly specific and sensitive diagnosis of gastric cancer. Further investigation revealed that serum DcR3 was positively associated with PDW in metastatic cancers and negatively associated with HGB and HCT in non-metastatic cancers. The combined detection of DcR3, PDW, HGB, and HCT acts as a better biomarker than DcR3 alone with respect to differentiating cancer metastasis from non-metastasis.

Numerous studies have shown DcR3 expression to be markedly increased in metastatic cancers, such as esophageal cancer [23], gastric cancer [24], and colon cancer [25]. In a recent study, we found that DcR3 promoted hepatoma cell migration by down-regulating E-cadherin expression [10]. E-cadherin, a classical member of the cadherin superfamily, is a calcium-dependent cell-cell adhesion glycoprotein that plays a key role in cellular adhesion [26, 27]. The loss of E-cadherin function or expression has been implicated in cancer progression and metastasis [28, 29]. In addition, DcR3 secreted by tumor cells is a negative regulator of MHC class II expression and a promoter of M2-like macrophage polarization (Tumour-Associated Macrophages promotion) [21, 30]. Moreover, DcR3 protects tumor cells from apoptosis and chemotaxis, which in turn results in a decreased immune response to the TH2 phenotype [31, 32]. The immune suppressing ability of DcR3 may facilitate cancer cell metastasis, which also partially explains the correlation between DcR3 and cancer metastasis.

Previous studies have indicated that platelet activation plays a key role in cancer metastases [33, 34]. Platelet activation refers to, at least partially abnormally increased PDW, a measure of platelet heterogeneity determined by heterogeneous demarcation of megakaryocytes [35]. Tumors could regulate megakaryocytic maturation, platelet production, and platelet size through interleukin-6 (IL-6), macrophage colony stimulating factor (M-CSF), and granulocyte colony-stimulating factor (G-CSF) [36]. One significant finding suggests that IL-6 secreted from tumor cells promotes tumorigenesis, angiogenesis, and metastasis [37]. Additionally, G-CSF and M-CSF secreted by tumor cells may stimulate megakaryopoiesis and subsequent thrombopoiesis [38]. These analyses may explain why higher DcR3 levels are accompanied by higher PDW in cancer metastasis.

The current study suggests that a higher DcR3 level in non-metastatic cancers is associated with both lower HCT and lower HGB, which indicate anemia in patients. High DcR3 levels are associated with cancer malignant progression, which is a consequence of complex interactions between the host microenvironment and tumor cells [39]. Anemia provides about 50–60% of local solid tumors with hypoxic tissue, which might produce more aggressive tumor clones [40]. But in our study, one difference is that serum DcR3 level is not correlated with HGB and HCT in cancers with metastasis. The disorders may be caused by the change of pre-metastatic niche [41]. The underlying mechanisms still remain unclear and need further investigation.

The study has two limitations. Firstly, from this study, 58 metastatic and 32 non-metastatic cases as well as 25 controls are included, which is a relatively small sample size. Secondly, the participants are all Chinese. Thus, further studies are needed to be conduct for other ethnic groups.

Our findings indicate that DcR3 is a potential biomarker for the detection of cancer cell metastasis across multiple cancer types; this biomarker could provide scientific clues for the evaluation and use of DcR3 in clinical diagnosis.

## MATERIALS AND METHODS

### Collection of clinical samples

The collection of human sera was approved by Ethics Committee of Shenzhen Second People's Hospital from January 2016 to December 2016. Informed consent was obtained from all patients and controls. All experiments using human blood samples were following the related technical and ethical guidelines. In our study, Sera from 90 tumor patients and 25 healthy individuals were collected during the same period. The mean age and age range at the time of diagnosis were 49 years and 24 to 92 years, respectively. Whole blood samples were collected in EDTA-containing tubes from the individuals with data on routine blood tests including platelet distribution width, hematocrit and hemoglobin, before initiating any treatment. All samples were processed within 30 min of blood collection.

### Reagents

The 96-well EIA/RIA high binding plates and bovine serum albumin (BSA) were bought from Sigma-Aldrich (Shanghai, China). Tris-buffered saline containing 0.05% Tween-20 (TBST), was acquired from double-helix (Shanghai, China). Tetramethylbenzidine dihydrochloride (TMB) and sulfuric acid were purchased from Sangon (Shanghai, China). Chinese hamster ovary cells, serum-free medium and spinner flasks were bought from Invitrogen (Carlsbad, CA, USA). The DcR3 ELISA kit was purchased from R&D (Minneapolis, MN, USA).

### Generation of monoclonal antibodies against human DcR3

Full-length human DcR3 cDNA fused to the sequence encoding the Fc domain of human IgG1 was here synthesized and cloned into a pcDNA3.1 expression vector (Invitrogen, Carlsbad, CA, USA). Chinese hamster ovary cells were transfected with this expression vector and grown in serum-free medium using a spinner flask. The soluble DcR3-Fc was purified from the medium and a panel of anti-DcR3 monoclonal antibodies against the recombinant protein was produced by hybridoma technology.

### Measurement of DcR3 using ELISA

We paired two antibodies, 5C3 and 4E7, to serve as capture and detection antibodies in double-antibody sandwiched ELISA. These antibodies both selectively recognized soluble DcR3 with higher sensibility and wider linear range than the DcR3 ELISA kit purchased from R&D (187–12000 pg/ml). Capture antibody was immobilized on the high binding plate. The plate was then blocked using 1% BSA. The plate was incubated with standards and serum samples at room temperature for 2 h, washed in TBST and incubated with detection antibody at room temperature for another 2 h. The plate was then sequentially incubated with streptavidin-HRP conjugations, TMB, and 1 mol/L of sulfuric acid. Data were recorded (at 450 nm) using a M1000 (Tecan) plate reader.

### Statistical analysis

Receiver operating characteristic (ROC) curve was used to analyze the classification power of candidate biomarkers. Pearson's test was used for correlation analysis between DcR3 and blood test indicators. The student's *t* test was used to evaluate the statistical significance of the difference in DcR3 between metastatic and non-metastatic cancers. The odds ratio (OR) and 95% confidence interval (95% CI) were calculated using multivariate logistic regression analysis. The statistical analysis was performed using GraphPad Prism version 5.0 for Windows (GraphPad Software, San Diego, CA, USA).

### Ethics section

The collection of human sera was approved by the Ethics Committee of Shenzhen Second People's Hospital. Informed consent was obtained from all patients and controls. All experiments using human blood samples were following the related technical and ethical guidelines.

## SUPPLEMENTARY MATERIALS FIGURES AND TABLE



## Abbreviations

DcR3: Decoy receptor 3
PDW: Platelet Distribution Width
HGB: Haemoglobin
HCT: Hematocrit
ROC: Receiver Operating Characteristic
TMB: Tetramethylbenzidine Dihydrochloride
OR: Odds Ratio
95% CI: 95% Confidence Interval
CEA: Carcino-Embryonic Antigen
AUC: Area Under Curve
