# Supplementary material for: DcR3 combined with hematological traits serves as a valuable biomarker for the diagnosis of cancer metastasis
Source: Oncotarget. 2017 Nov 20;8(64):107612–20. doi: 10.18632/oncotarget.22544 (PMC5746094; doi:10.18632/oncotarget.22544)
Supplement: Supplementary file 1 [file oncotarget-08-107612-s001.pdf]

DcR3 combined with hematological traits serves as a valuable biomarker for the diagnosis of cancer metastasis

SUPPLEMENTARY MATERIALS

Supplementary Table 1: Metastasis risks according to combination of DcR3 and hematological traits

Parameter	DcR3 × PDW			DcR3 ÷ (HGB × HCT)			DcR3 ÷ HGB			DcR3 ÷ HCT		
	>2100.5	<2100.5	OR (95% CI) *P*-value	>0.1	<0.1	OR (95% CI) *P*-value	>1.6	<1.6	OR (95% CI) *P*-value	>5.4	<5.4	OR (95% CI) *P*-value
Metastasis (*n* = 58)	*n* = 46	*n* = 12	6.39 (2.45–16.75)	*n* = 19	*n* = 39	15.10 (1.91–119.14)	*n* = 44	*n* = 14	4.59 (1.82–11.61)	*n* = 45	*n* = 13	5.76 (2.24–14.84)
Non-Metastasis (*n* = 32)	*n* = 12	*n* = 20	0.0001	*n* = 1	*n* = 31	0.01	*n* = 13	*n* = 19	0.0013	*n* = 12	*n* = 20	0.0003

The number of patients tested (*n*) is shown.
OR: Odds Ratio.
CI: Confidence Interval.

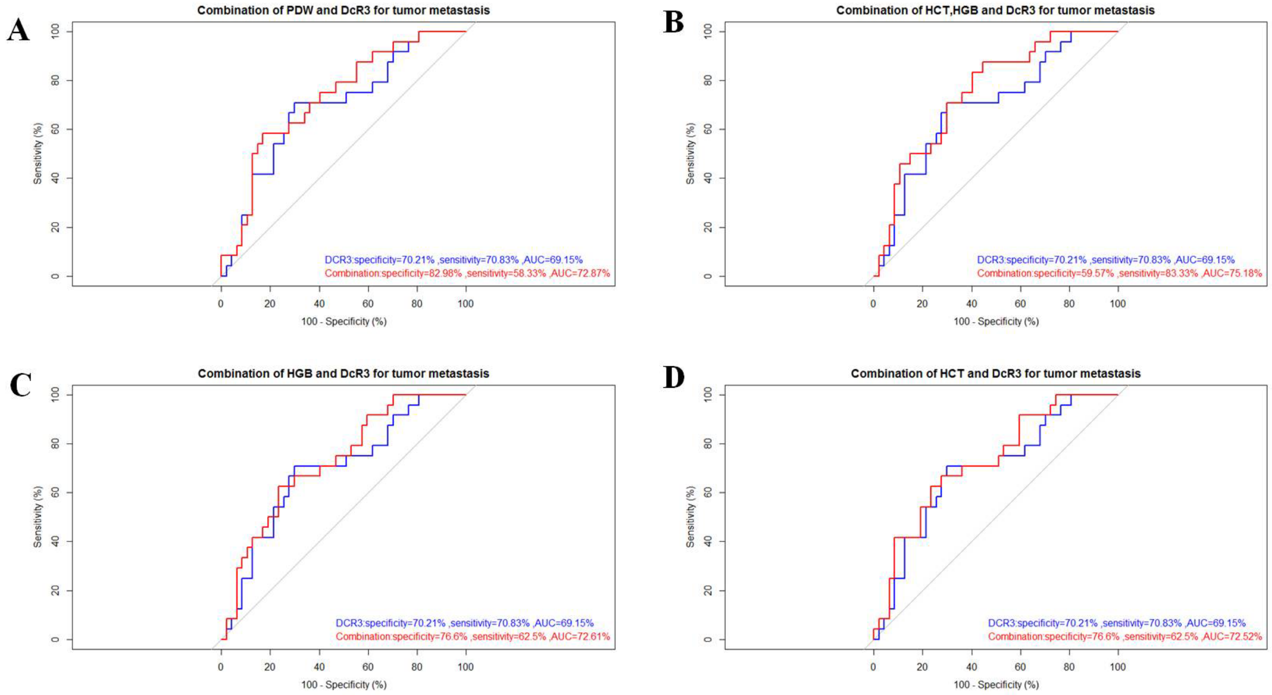

Supplementary Figure 1: ROC curve showed the utility of alone or combination for the diagnosis of tumor metastasis. Equation 2 = DcR3 × PDW, Equation 3 = DcR3 ÷ (HGB × HCT), Equation 4 = DcR3 ÷ HGB, Equation 5 = DcR3 ÷ HCT. (**A**) ROC curve showed the utility of Equation 2 for the diagnosis of tumor metastasis. sensitivity = 82.98%, specificity = 58.33%, AUC = 72.87%, threshold = 2100.5. (**B**) ROC curve showed the utility of Equation 3 for the diagnosis of tumor metastasis. sensitivity = 59.57%, specificity = 83.33%, AUC = 75.18%, threshold = 0.1. (**C**) ROC curve showed the utility of Equation 4 for the diagnosis of tumor metastasis. sensitivity = 76.6%, specificity = 62.5%, AUC = 72.61%, threshold = 1.6. (**D**) ROC curve showed the utility of Equation 5 for the diagnosis of tumor metastasis. sensitivity = 76.6%, specificity = 62.5%, AUC = 72.52%, threshold = 5.4.

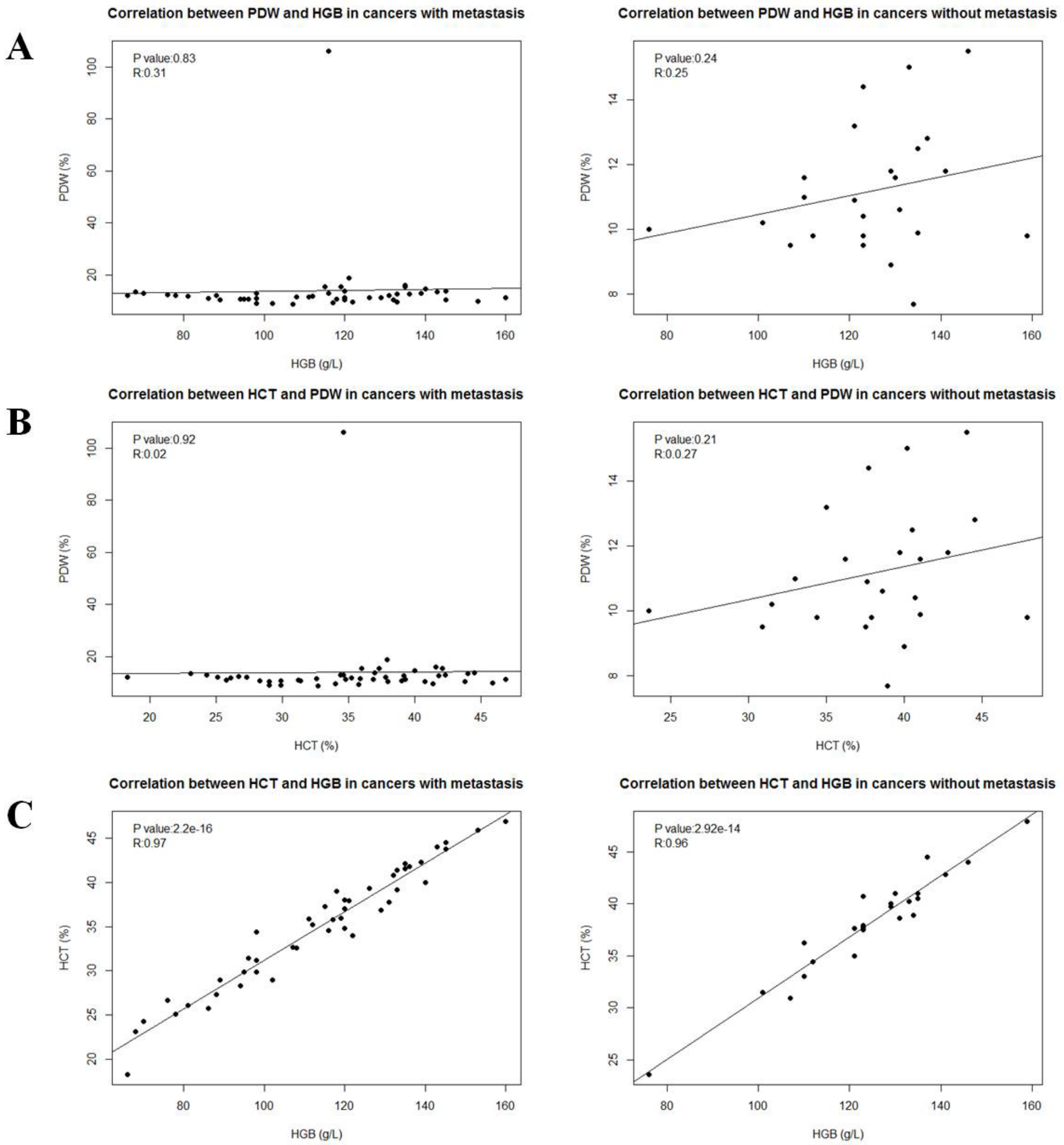

Supplementary Figure 2: Correlations among hematological traits. (**A**) PDW was not correlated with HGB in cancers with (R = 0.31, P = 0.83) and without (R = 0.25, P = 0.24) metastasis. (**B**) PDW was not correlated with HCT in cancers with (R = 0.02, P = 0.92) and without (R = 0.027, P = 0.21) metastasis. (**C**) HCT was positively correlated with HGB in cancers with (R = 0.97, $P = 2.2 \times 10^{-16}$) and without (R = 0.96, $P = 2.92 \times 10^{-14}$) metastasis.
